# Comparative Transcriptome Profiles of the Response of Mycelia of the Genus *Morchella* to Temperature Stress: An Examination of Potential Resistance Mechanisms

**DOI:** 10.3390/jof10030178

**Published:** 2024-02-27

**Authors:** Yihong Yue, Haibo Hao, Qian Wang, Tingting Xiao, Yuchen Zhang, Hui Chen, Jinjing Zhang

**Affiliations:** 1 National Research Center for Edible Fungi Biotechnology and Engineering, Key Laboratory of Applied Mycological Resources and Utilization, Ministry of Agriculture, Shanghai Key Laboratory of Agricultural Genetics and Breeding, Institute of Edible Fungi, Shanghai Academy of Agricultural Sciences, Shanghai 201403, China; yihongyue@saas.sh.cn (Y.Y.); hb199232@163.com (H.H.); wq-15309@163.com (Q.W.); xiaotingting@saas.sh.cn (T.X.); zyc0216@saas.sh.cn (Y.Z.); 2 State Key Laboratory of Genetic Engineering and Fudan Center for Genetic Diversity and Designing Agriculture, Institute of Plant Biology, School of Life Sciences, Fudan University, Shanghai 200438, China

**Keywords:** antioxidant enzyme, energy metabolism, *Morchella* mycelium, purine metabolism, temperature stress, transcriptomic analysis

## Abstract

Temperature and moisture belong to the most important environmental factors affecting the growth and development of fungi. However, the effect of temperature on the mycelia of the edible *Morchella* mushrooms has not been determined. Here, a comprehensive analysis was performed to determine the influence of culture temperature on 13 strains of mycelia of three *Morchella* species (*Morchella sextelata*, *Morchella septimelata*, and *Morchella importuna*) at 5 °C, 10 °C, 15 °C, 20 °C, 25 °C, and 30 °C. The mycelial branching and growth rate data showed that 15–20 °C was a suitable temperature range for the mycelial growth of the 13 *Morchella* strains. RNA sequences revealed that a total of 2843, 2404, 1973, 1572, and 1866 differentially expressed genes (DEGs) were identified at 5 °C, 10 °C, 15 °C, 25 °C, and 30 °C compared with 20 °C. A Kyoto Encyclopedia of Genes and Genomes (KEGG) enrichment analysis further indicated that the purine nucleotide and tyrosine metabolism pathways were crucial for mycelium development. Moreover, the enrichment of autophagy of mitochondria, regulation of cell morphogenesis, and piecemeal microautophagy of the nuclei at 25 °C (vs. 20 °C) indicated the damage caused by heat stress in *Morchella* mycelia. Notably, a total of four unique module eigengenes (MEs) were identified through a weighted gene coexpression network analysis (WGCNA). Among them, 2293 genes in the turquoise module were significantly positively correlated with temperature (r = 0.946, *p* < 0.001), whereas 739 genes in the blue module were significantly negatively correlated with temperature (r = −0.896, *p* < 0.001), suggesting that the effect of high temperatures on mycelial genes was significantly greater than that of low temperatures. Moreover, the coexpression network indicated that high culture temperatures accelerated the oxidative stress response and energy metabolism in mycelia, while upregulation of purine nucleotide catabolism and ribosomal protein-related genes were improved by low-temperature tolerance. In addition, the upregulated expression of superoxide dismutase (SOD), catalase (CAT), glutathione peroxidase (GPX), and heat shock protein (HSP) genes in mycelia was associated with reactive oxygen species (ROS)-mediated damage at high temperatures. Overall, this study provides an important theoretical basis and application value for optimizing *Morchella* cultivation techniques.

## 1. Introduction

Acscomycetes morels (*Morchella* spp.) are rare wild edible mushrooms rich in amino acids and flavoring substances [1,2]. Their notable nutritional value and delicious taste make them one of the most popular edible fungi worldwide [3]. Moreover, *Morchella* species possess important economic and medicinal value because of their antioxidant [4], anti-inflammatory [5], antibacterial [6], antitumor [7], and immunomodulatory [8] effects through their abundant bioactive compounds. The domestication and cultivation of valuable *Morchella* fungi have a history spanning more than 130 years, but artificial cultivation of these species is still associated with unstable yields [9,10]. Edible *Morchella* mushrooms can be cultivated at low temperatures. Due to temperature stress, artificial cultivation in various regions in recent years has caused problems such as a decrease in or even absence of *Morchella* harvests.

In agricultural production, temperature is one of the most important environmental factors affecting the artificial cultivation of edible fungi [11,12]. Unfavorably high ambient temperatures significantly affect the activity of substrate-degrading enzymes and antioxidant enzymes in mycelia during the nutritional growth stage, alter the conversion rate of proteins and polysaccharides, and cause differences in material conditions during primordial differentiation, thereby triggering a series of physiological and biochemical responses [13]. Both cold stress and heat stimulation can lead to the excessive accumulation of reactive oxygen species (ROS), which oxidize large molecules such as DNA, lipids, and proteins, causing oxidative damage in fungi [14]. Edible fungi produce different functional proteins to cope with temperature stress and alleviate damage. Previous research has shown that, at the vegetative growth stage, *Pleurotus ostreatus* exhibits the highest activities of superoxide dismutase (SOD), catalase (CAT), and ascorbate peroxidase (APX) at high temperatures [15], while *Stropharia rugosoannulata* reduces oxidative damage caused by low temperatures through high expression of antioxidant enzymes [16]. In addition, heatshock proteins (HSPs) and their cognates, as primary mitigators of cell stress, are significantly upregulated to reshape stress-induced cellular functional damage and protein misfolding, enhancing fungal temperature tolerance [17,18,19]. Hence, understanding the relationships between mycelial growth and temperature stress is valuable.

Research on the genetic basis of the low-temperature preference of morels is crucial for germplasm innovation. Unlike other mushroom cultivation methods, the artificial cultivation of morels involves the use of cultured mycelia directly sown in the soil. Mycelial growth requires the maintenance of a low temperature for 20–30 days during the overwintering process, followed by an increase in temperature to stimulate the differentiation of the primordia into fruiting bodies, transforming vegetative growth into reproductive growth [20]. In artificial cultures of several other edible mushrooms growing on wood chips (e.g., Shii-take, *Lentinula edodes*), the contrary is true: a higher temperature for the colonization of the substrate (24–28 °C) is followed by lower temperatures (12–20 °C) for fruit-body development [21]. Comprehensive transcriptomic analysis of *Morchella* revealed that carbohydrate catabolism occurs mainly during the vegetative mycelium stage [22], with starch and sucrose metabolism, pentose and glucuronate interconversions, fructose, mannose, tyrosine, and purine metabolisms being key pathways involved in the development of *Morchella* mycelia [23]. The expression levels of genes encoding carbohydrate-active enzymes (CAZymes), mitochondrial proteins, oxidoreductases, and heat shock proteins in *Morchella* mycelia were significantly lower than those in young fruiting bodies [20]. However, the exact effects and molecular mechanisms underlying *Morchella* mycelial responses to adverse temperature stress during the vegetative growth period have not been fully elucidated, which may limit the further industrial cultivation of *Morchella* species [24]. It has been reported that insufficient nutrition at low or high temperatures may cause severe disease with red-stipe symptoms, resulting in growth cessation and rot of the fruiting body [25]. Therefore, elucidating the mechanism underlying the effect of temperature on mycelia is an important prerequisite for achieving stable and high yields of *Morchella* and has important guiding significance and application value for optimizing cultivation techniques.

In this study, to further systematically determine the molecular mechanism of mycelial growth in the tree *Morchella* species *M. sextelata*, *M. septimelata*, and *M. importuna*, transcriptome sequencing was performed at culture temperatures of 5 °C, 10 °C, 15 °C, 20 °C, 25 °C, and 30 °C to investigate the crucial genes and pathways involved in vegetative growth. This study may help to elucidate the metabolic mechanisms involved in the mycelial growth of *Morchella* and provide a valuable genetic basis for improving productivity during *Morchella* cultivation.

## 2. Materials and Methods

### 2.1. Fungal Strain and Culture Conditions

The 13 *Morchella* strains used in this study originated from the Hebei, Hubei, Shanxi, Sichuan, and Qinghai provinces, China, and were preserved at the Institute of Edible Fungi, Shanghai Academy of Agricultural Sciences, Shanghai, China. Detailed information on the *Morchella* strains is provided in Table S1. The *Morchella* strains were grown in PDA medium supplemented with 200 g/L potato extract, 20 g/L glucose, 2 g/L KH_2_PO_4_, 2 g/L peptone, 1 g/L MgSO_4_, and 2.2 g/L agar at 5 °C, 10 °C, 15 °C, 20 °C, 25 °C, and 30 °C in a constant-temperature incubator. During the mycelial growth process, the diameters of the mycelia extending toward the plate were recorded after 24 and 48 h of cultivation to calculate the mycelial growth rate in each treatment group. Moreover, hyphal branching was observed using an inverted microscope (Zeiss LSM880, Carl Zeiss AG, Oberkochen, Germany). After five days of cultivation in the dark, the mycelia were collected in sterile bags with a clean nipper. Three samples from each treatment group were analyzed in parallel. Subsequently, the obtained mycelia were completely dried with filter paper and stored at −80 °C for transcriptomic sequencing.

### 2.2. RNA Extraction, Library Preparation, and RNA Sequencing

Using TRIzol^®^ Reagent (Invitrogen, Carlsbad, CA, USA), total RNA was extracted from the mycelia according to the manufacturer’s instructions. RNA quantity and quality were measured using a Qubit 2.0 Fluorometer (Thermo Scientific, Waltham, MA, USA) and an Agilent 2100 Bioanalyzer (Agilent, Palo Alto, CA, USA) with the following standards: OD260/280 = 1.8–2.2, OD260/230 ≥ 2.0, RIN ≥ 6.5, and 28S:18S ≥ 1.0. The library was prepared using the TruSeq™ RNA sample preparation kit (Illumina, San Diego, CA, USA) and further sequenced on a NovaSeq 6000 (Illumina, San Diego, CA, USA) platform with a 2 × 150 bp read length by Shanghai Biozeron Biotechnology Co., Ltd. (Shanghai, China).

### 2.3. Data processing and Bioinformatics Analysis

The raw data were trimmed and quality controlled through the removal of sequence adaptors, reads with quality under Q20, and reads with less than 50 bases. The high-quality sequences were mapped to the *Morchella sextelata* genome (ASM2013738v1, https://www.ncbi.nlm.nih.gov/datasets/genome/GCF_020137385.1/, accessed on 24 September 2021) by Hierarchical Indexing for Spliced Alignment of Transcripts (HISAT2, v2.0.5). The mapped reads of each sample were assembled by StringTie. Subsequently, the expression levels of genes in each sample were calculated through the fragments per kilo base of exon per million mapped reads (FRKM) method, and the results were subsequently used to compare gene expression differences between samples and identify corresponding differentially expressed genes (DEGs) via the R statistical package software EdgeR (Empirical Analysis of Digital Gene Expression in R, v3.6.3). The screening criteria for identifying significant DEGs were |log2 (FC)| ≥ 1 and FDR ≤ 0.05. Furthermore, functional annotation was carried out via the Gene Ontology (GO) database and the Kyoto Encyclopedia of Genes and Genomes (KEGG) database. GO functional enrichment and KEGG pathway analyses were performed using Goatools (v0.9.9) and KOBAS (v3), respectively.

### 2.4. Statistical Analysis

To measure the degree of correlation between samples, the Pearson method was used to conduct a correlation analysis on each sample based on gene expression levels, and the results are presented in a heatmap. A principal component analysis (PCA) was used to evaluate the overall differences in expression between groups and the degree of variation between samples within the groups. A gene set enrichment analysis is a computational method that was used to compare 5 °C-, 10 °C-, 15 °C-, 25 °C-, and 30 °C-treated and 20 °C-treated mycelia. The list of genes of interest was first imported into the GSEA software (v4.3.0), after which we determined whether an a priori defined set of genes showed statistically significant concordant differences between the two treatment groups. The *p* value was calculated using a bootstrap distribution created by resampling a set of genes with the same cardinality. A weighted gene correlation network analysis (WGCNA) was performed to analyze the relationships and networks involving the various genes in the transcriptome. To construct a scale-free network, an adjacency function from coexpression data in the WGCNA was used to weigh different genes using aij = (Sij, β) = |Sij|^β^. The weighted parameter power beta value was determined from the scale-free topology criterion to ensure that the average connectivity of the network was smooth. Moreover, functional modules with different colors were obtained using the step-by-step network construction function on blockwise modules with default settings. The topological overlap matrix (TOM) type was set to signed, the min module size was set to 30, and the merge cut height was set to 0.25. A heatmap of the correlations between the modules and phenotypes was drawn to determine which modules were strongly correlated with the phenotypes, and then the gene modules were further associated with the phenotypes. The networks were constructed and visualized using the Cytoscape (v3.5.1) software.

### 2.5. Enzyme Assay

The mycelial samples from each treatment group were ground into a fine powder in liquid nitrogen. Then, 0.1 g of the powder was placed in 1 mL of extraction solution for homogenization in an ice bath and centrifuged at 9000× *g* for 10 min at 4 °C. The supernatant was collected and analyzed with a corresponding detection kit (Keming Biotechnology, Suzhou, China) to measure the activities of superoxide dismutase (SOD) and glutathione (GSH) (Comin Biotechnology, Suzhou, China). For each sample, three parallel experiments were performed.

### 2.6. Data Availability

The raw reads obtained from Illumina NovaSeq in this study have been deposited into the NCBI Sequence Read Archive (SRA) database under accession number PRJNA1056473.

## 3. Results

### 3.1. Effects of Culture Temperature on the Mycelial Growth of Morchella

Mycelial growth significantly varied among the *Morchella* species cultured at different temperatures (Figure S1). Morphologically, 13 *Morchella* strains (10 strains of *M. sextelata*, 2 strains of *M. septimelata*, and 1 strain of *M. importuna*) had thin and sparse mycelia at 5 °C, which gradually thickened at 10 °C (Figure S1A). The mycelia were white, dense, and robust and had neat tips when cultured at 15–20 °C. When the culture temperature was 25 °C, the mycelia began to age, and yellow-brown mycelia appeared. The mycelial morphology at 30 °C was abnormal, and the mycelia appeared dark brown, exhibiting significantly inhibited growth. Moreover, the number of pronged branches of *Morchella* mycelia increased with increasing temperature (Figure S1B). At 5 °C, the hyphae were relatively loose and straight, with shorter and fewer pronged branches; at 10–20 °C, the number of pronged branches and the density of the mycelia gradually increased. The highest number of pronged branches and the thickest mycelia occurred at 25 °C, indicating that the mycelia rapidly bifurcated to adapt to the temperature increase. When the culture temperature was 30 °C, clear mycelial bending and twisting were observed. This variation was consistent with the mycelial morphology. These results suggested that a culture temperature above 20 °C was not suitable for the mycelial growth of *Morchella* and that the mycelial morphology improved below 20 °C.

**Figure 1:**
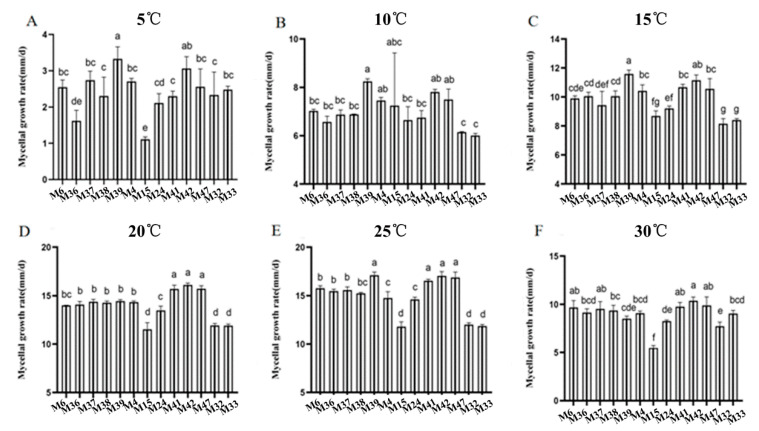
Comparison of mycelial growth rates of *Morchella* strains cultured at 5 °C (**A**), 10 °C (**B**), 15 °C (**C**), 20 °C (**D**), 25 °C (**E**), and 30 °C (**F**). Numbers in a rank with different letters indicate significant differences (Tukey’s HSD test, *p* < 0.05).

To quantify the effects of culture temperature on vegetative growth, the mycelial growth rate of *Morchella* was measured after 3 days of culture (Figure 1). Among the thirteen *Morchella* strains, M39 was the fastest-growing strain at 5 °C, 10 °C, and 15 °C, while M42 grew the fastest at culture temperatures of 20 °C, 25 °C, and 30 °C. M15, M32, and M33 belonged to *M. importuna* (M. Kuo, O’ Donnell & T.J.Volk) and *M. septimelata* (M. Kuo) and had the slowest growth rates at all the culture temperatures, indicating that the mycelial growth rate varied among the different varieties of *Morchella*. With increasing temperature, the mycelial growth rate first increased and then decreased (Table S2). The fastest mycelial growth rate occurred at 20 °C and 25 °C, with 10 out of the 13 strains achieving a growth rate of 13.4 mm/d, except for *M. importuna* and *M. septimelata*, while the mycelial growth rate decreased significantly at 30 °C (*p* < 0.05). By comprehensive comparison, a temperature range of 15–20 °C was determined to be suitable for the mycelial growth of *Morchella*.

### 3.2. Overview of the Transcriptomic Analysis

To obtain an overview of mycelial gene expression at different culture temperatures, the M37 strain, as a representative of *M. sextelata*, was selected for transcriptome sequencing (Table S3). A total of 805,074,976 high-quality clean reads were generated from 18 cDNA libraries. The Q30 value of the base ratio was greater than 91.3%. The *M. sextelata* genome (ASM2013738v1, https://www.ncbi.nlm.nih.gov/datasets/genome/GCF_020137385.1/, accessed on 24 September 2021) was selected for sequence alignment. A cluster analysis of the whole genome showed that the *M. sextelata* (M. Kuo) mycelia cultured at 5 °C, 10 °C, 15 °C, 20 °C, 25 °C, and 30 °C were well separated (Figure S2A). Similarly, PCA also revealed significant intergroup differences among the treatment groups as the culture temperature increased, especially at 20 °C, 25 °C, and 30 °C and 5 °C, 10 °C, and 15 °C (Figure S2B). Therefore, mycelia cultured at 20 °C were selected as control groups for subsequent analysis.

### 3.3. Identification of Expression Levels and Differentially Expressed Genes (DEGs)

With |log2 FC| ≥ 1 and *p* < 0.05 as the thresholds for FPKM (fragments per kilobase per million reads) values, the genes differentially expressed at different culture temperatures were identified (Figure S3). Among the differentially expressed genes (DEGs), 2843, 2404, 1973, 1572, and 1866 were differentially expressed at 5 °C, 10 °C, 15 °C, 25 °C, and 30 °C, respectively (vs. 20 °C) (Figure S3A). For the comparisons of 5 °C, 10 °C, 15 °C, 25 °C, and 30 °C vs. 20 °C, 785, 800, 732, 843, and 1281 upregulated DEGs, respectively, and 2058, 1604, 1241, 729, and 585 downregulated DEGs, respectively, were obtained (Figure S3B). When the temperature exceeded 20 °C, the number of upregulated DEGs increased significantly, while the number of downregulated DEGs decreased sharply, indicating that high temperatures strongly influenced mycelial growth.

### 3.4. Functional Annotation and Enrichment Analysis of DEGs

**Figure 2:**
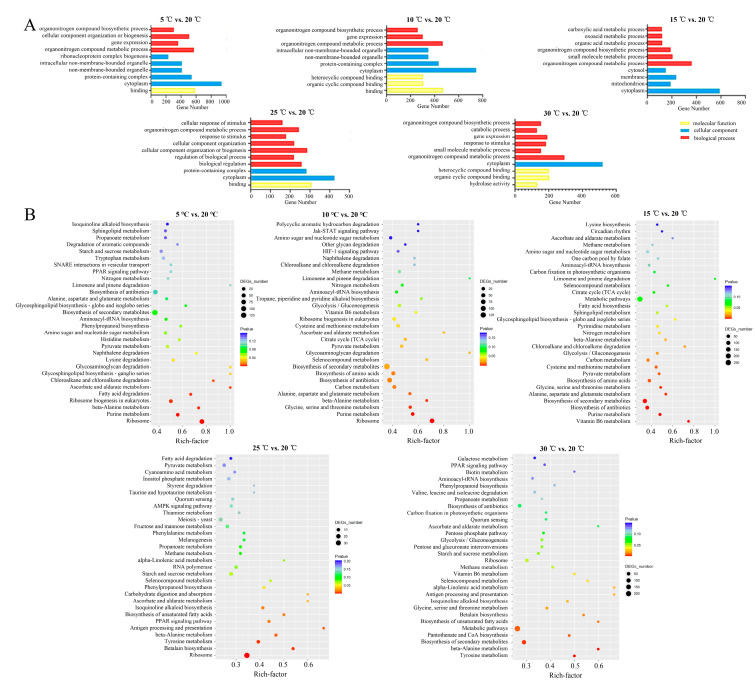
The most enriched Gene Ontology (GO) (**A**) and KEGG (**B**) annotations of DEGs in the five comparison groups. BP, biological process; CC, cellular component; MF, molecular function.

To investigate the biological processes associated with the DEGs at different culture temperatures, the most enriched Gene Ontology (GO) terms were identified with the criterion *p* ≤ 0.05 in the five comparison groups (Figure 2A). Among these, “cytoplasm” (cellular component) and “organonitrogen compound metabolic process” (biological process) were the most common in the five comparison groups. Moreover, “binding” in the molecular function category in the 5 °C-vs.-20 °C, 10 °C-vs.-20 °C, and 25 °C-vs.-20 °C comparisons was the most significantly enriched, while “heterocyclic compound binding” and “organic cyclic compound binding” in the molecular function category in the 30 °C-vs.-20 °C comparison group were the most significantly enriched.

To further analyze the DEG-related pathways involved in mycelial development at different culture temperatures, the top 30 most highly enriched pathways in each comparison group were selected based on the rich factor and *p* value (Figure 2B). The ribosome, purine metabolism, and beta-alanine metabolism (ko00410) pathways were common to the 5 °C-vs.-20 °C and 10 °C-vs.-20 °C comparisons. Vitamin B6 metabolism, biosynthesis of antibiotics, and biosynthesis of secondary metabolites were the most significantly enriched pathways in the 15 °C-vs.-20 °C comparison. Ribosome, tyrosine metabolism, and betalain biosynthesis were the most significantly enriched pathways in the 25 °C-vs.-20 °C comparison, while tyrosine metabolism, beta-alanine metabolism, and metabolic pathways were the most significantly enriched in the 30 °C-vs.-20 °C comparison.

### 3.5. Gene Set Enrichment Analysis

**Figure 3:**
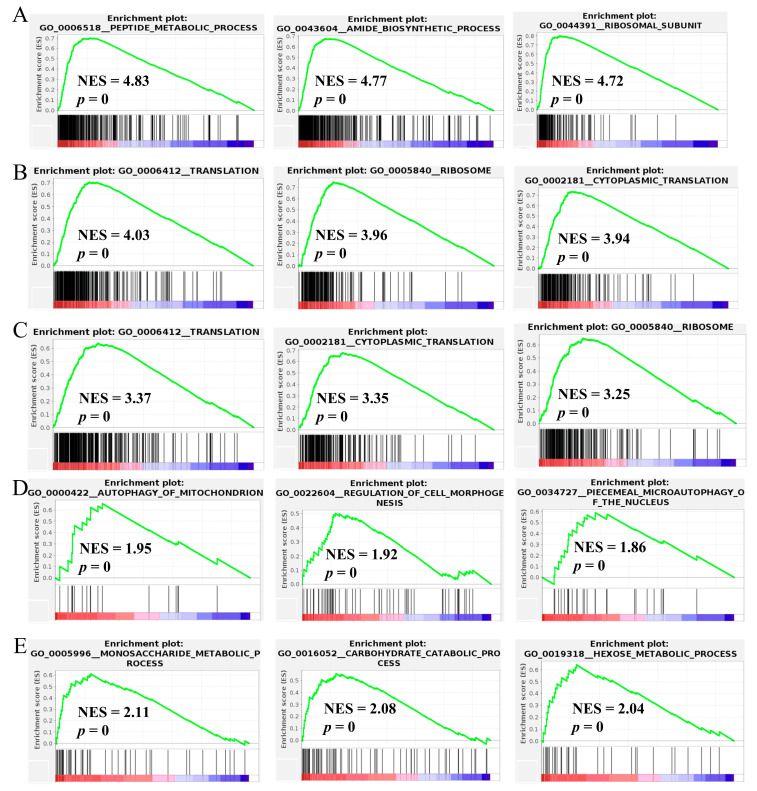
Gene set enrichment analysis (GSEA) at culture temperatures of 5 °C (**A**), 10 °C (**B**), 15 °C (**C**), 25 °C (**D**), and 30 °C (**E**) (vs. 20 °C).

To determine the potential mechanism through which culture temperature-related hub DMGs are involved in mycelial growth, a gene set enrichment analysis (GSEA) was conducted (Table 1 and Figure 3). At 5 °C (vs. 20 °C), the peptide metabolic process, the amide biosynthetic process, and ribosomal subunits were enriched. At 10 °C (vs. 20 °C), translation, ribosomes, and cytoplasmic translation were enriched. At 15 °C (vs. 20 °C), translation, cytoplasmic translation, and ribosomes were enriched. At 25 °C (vs. 20 °C), autophagy of mitochondria, regulation of cell morphogenesis, and piecemeal microautophagy of the nucleus were enriched. At 30 °C (vs. 20 °C), monosaccharide metabolic, carbohydrate catabolic, and hexose metabolic processes were enriched.

### 3.6. Weighted Coexpression Network Construction and Identification of Module Eigengenes

**Figure 4:**
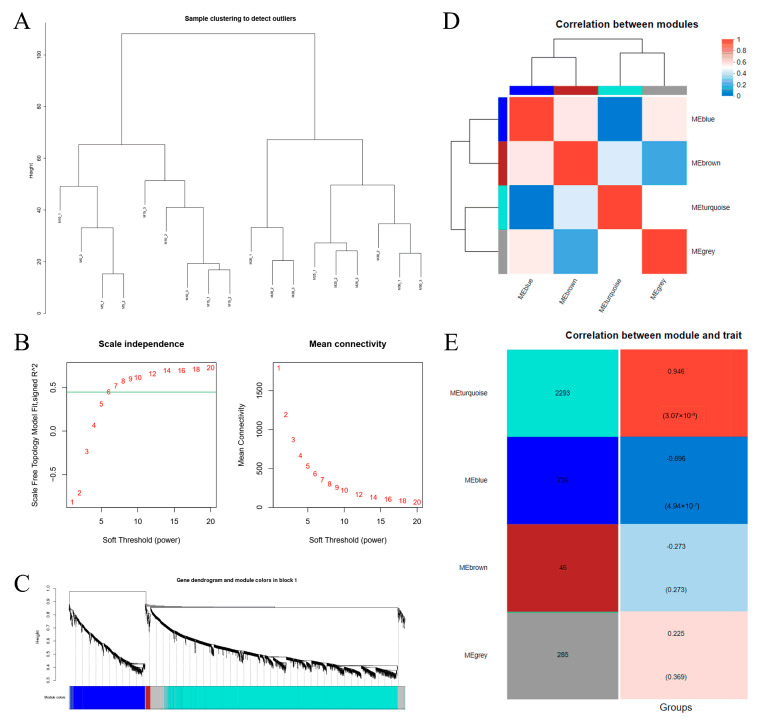
Weighted gene coexpression network analysis (WGCNA). (**A**) Clustering dendrogram of 18 samples. (**B**) Analysis of scale-free fit indices for various soft-threshold powers (β) and the mean connectivity for various soft-threshold powers to determine soft-threshold power. (**C**) Dendrogram of all expressed genes in the top 25% in terms of variance, clustered based on a dissimilarity measure (1 − TOM). (**D**) Heatmap based on the module eigengenes. (**E**) Heatmap of the correlation between the ME and temperature module. TOM, topological overlap matrix; MEs, module eigengenes.

Using a weighted gene coexpression network analysis (WGCNA), a coexpression network analysis was conducted on unigenes from mycelia cultured at different temperatures to explore the relationships and networks between various genes (Figure 4). Hierarchical clustering of the RNA sequence data of 18 samples revealed greater similarity among the transcriptomes obtained at culture temperatures of 5 °C, 10 °C, and 15 °C and those obtained at 20 °C, 25 °C, and 30 °C (Figure 4A), which was consistent with the PCA results (Figure S2B). To ensure that the average connectivity of the network was smooth, the final optimal value of thresholding power was β = 6 (Figure 4B). Subsequently, a total of four unique module eigengenes (MEs) were identified (Figure 4C,D). The ME of the turquoise module, which contained 2293 genes, was significantly positively correlated with culture temperature (r = 0.946, *p* < 0.001), while the ME of the blue module, which contained 739 genes, was significantly negatively correlated with culture temperature (r = −0.896, *p* < 0.001) (Figure 4E). These findings suggested that the effect of high temperatures on mycelial genes was significantly greater than that of low temperatures.

### 3.7. Functional Enrichment Analyses of Genes in the ME Turquoise and Blue Modules

**Figure 5:**
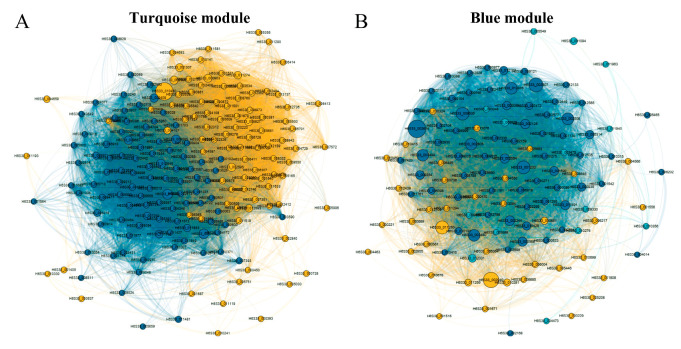
Coexpression networks of genes in the ME turquoise (**A**) and blue (**B**) modules.

To understand the influence of culture temperature on mycelial growth, the ME turquoise and blue modules were focused on in this study (Figure 5). The network in the ME turquoise module had 11,940 edges with 194 nodes connected (Figure 5A), whereas that in the ME blue module had 4710 edges with 122 nodes (Figure 5B). All nodes in the ME turquoise and blue modules were classified into two and three key modules, respectively. For the ME turquoise module, module I gene functions were primarily enriched for oxidative dehydrogenation, including alpha-ketoacid dehydrogenase kinase (H6S33_000063), sphingolipid fatty acid hydroxylase (H6S33_000109), mitochondrial dicarboxylate carrier protein (H6S33_000464), putative Fe-containing alcohol dehydrogenase (H6S33_000501), aldehyde dehydrogenase (H6S33_000904, H6S33_011849), oxidoreductase (H6S33_011977), and pyruvate decarboxylase (H6S33_012945). The functions of the genes in module II were related to the catabolic process of glucose with putative MFS monosaccharide transporter (H6S33_000450), alpha/beta-hydrolase (H6S33_000895), glycoside hydrolase/deacetylase (H6S33_005331, H6S33_012593), and glyceraldehyde 3-phosphate dehydrogenase (H6S33_011274). Moreover, two cytochrome-related genes, H6S33_008973 and H6S33_007572, were identified. These results indicated that high culture temperatures accelerated the oxidative stress response, energy metabolism, and cytochrome accumulation in mycelia.

For the ME blue module, many ribosomal protein-related genes in module I were enriched, including ribosomal protein L13e (H6S33_000091), 60S ribosomal protein L5 (H6S33_009104), 60S ribosomal protein L22 (H6S33_009354), ribosomal protein S12/S23 (H6S33_009535), pre-rRNA-processing protein PNO1 (H6S33_009546), mitochondrial ribosomal protein L44 (H6S33_010275), ribosomal protein S3 (H6S33_012107), ribosomal protein L28e (H6S33_012535), ribosomal protein L35Ae (H6S33_001200), ribosomal protein 60S (H6S33_002125), ribosomal protein S28e (H6S33_002244), putative 60S ribosomal protein L21-B (H6S33_002507), ribosomal protein S5 (H6S33_002554), ribosomal protein S8e (H6S33_002577), putative 40S ribosomal protein S22 (H6S33_002614), ribosomal protein S9 (H6S33_002805), ribosomal protein S7e (H6S33_002930), 60S ribosomal protein L20 (H6S33_003480), and ribosomal protein L38e (H6S33_005098). Moreover, genes involved in the catabolism of purine nucleotides, such as xanthine dehydrogenase (H6S33_000221), S-methyl-5’-thioadenosine (H6S33_000470), hypoxanthine guanine phosphoribosyl transferase (H6S33_000561), and phospholipase C/P1 nuclease (H6S33_006004), were enriched in module II. Notably, H6S33_001094, a flavoprotein-related protein, was identified in module III.

### 3.8. Effects of Mycelial Culture Temperature on the Gene Expression of Antioxidant Enzymes and the Ubiquitin–Proteasome System

**Figure 6:**
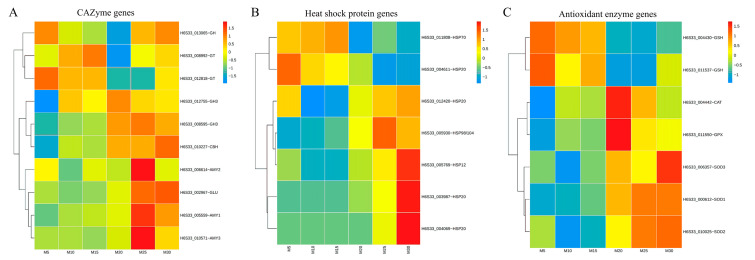
Analysis of CAZyme gene (**A**), antioxidant enzyme (**B**), and heat shock protein (**C**) expression in *Morchella* mycelia under different temperature treatments.

To comprehensively analyze the expression patterns of CAZyme genes, heat shock proteins, and antioxidant enzymes in *Morchella* mycelia at different temperatures, the top ten, seven, and seven genes, respectively, with significant changes in expression were screened for analysis (Figure 6). Among the CAZyme genes, amylases (amy1, amy2, and amy3) and glucosidase (glu) were highly expressed at 25 °C and 30 °C, while glycosyl hydrolases (GH3) and cellobiohydrolase (CBH) were highly expressed at 20–30 °C, whereas glycosyl transferases (GTs) were highly expressed at 5–15 °C and 25–30 °C (Figure 6A). Among the heat shock proteins, HSP20 (H6S33-004069, H6S33-003987, and H6S33-012420), HSP12 (H6S33-005769), and HSP98/104 (H6S33-005930) were upregulated at 25 °C and 30 °C, while HSP20 (H6S33-004611) and HSP70 (H6S33-011808) were upregulated at 5–15 °C (Figure 6B). Among the antioxidant enzyme-encoding genes, SOD genes (SOD1, SOD2, and SOD3) were highly expressed at 25 °C and 30 °C, while CAT and glutathione peroxidase (GPX) genes were highly expressed at 20 °C and 25 °C, whereas the reduced glutathione gene (gsh) was highly expressed at 5–15 °C (Figure 6C). These results indicated that the damage caused by high-temperature stress on the mycelia of *Morchella* was more severe than that caused by low-temperature stress.

**Figure 7:**
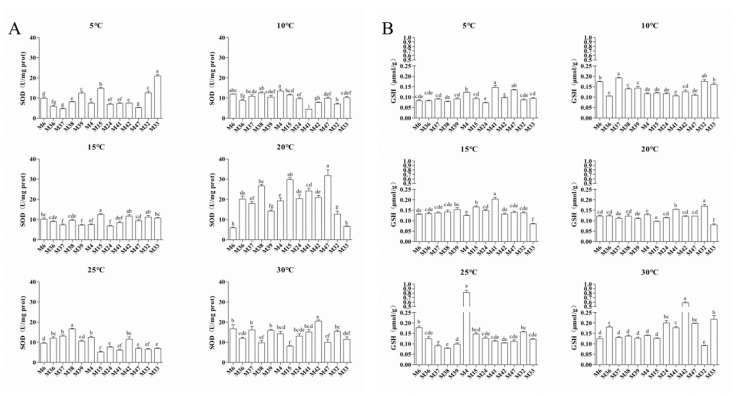
Analysis of SOD (**A**) and GSH (**B**) activities in 13 *Morchella* strains under different temperature treatments. The error bars represent the means ± standard deviations of triplicate experiments.

The antioxidant enzyme activity was further measured in 13 strains of Morchella under different culture temperatures (Figure 7). The SOD enzyme activity of 11 strains of *M. sextelata* and *M. importuna* was significantly greater at 20–30 °C than at 5–15 °C, while the M33 strain of *M. septimelata* had the highest SOD activity at 5 °C (Figure 7A). Similarly, the GSH of 13 strains presented the highest activity at 20 °C and 30 °C, while the GSH activity of the M4 strain peaked at 25 °C (Figure 7B). These results illustrated that the mycelia might be more susceptible to high-temperature stress than low-temperature damage.

According to functional annotations, 22 genes related to the ubiquitin–proteasome system were detected, and the top 6 genes were analyzed (Figure S4). With the increase in temperature, the expression levels of ubiquitin-40S ribosomal protein S27a (H6S33_013100) and ubiquitin-60S ribosomal protein L40 (H6S33_011254) significantly decreased (*p* < 0.05), while the gene of ubiquitin-conjugating enzyme (H6S33_000510) significantly increased (*p* < 0.05). Meanwhile, polyubiquitin-tagged protein recognition complex and Npl4 component (H6S33_001104) were upregulated at 5–15 °C, while ubiquitin-related domain-containing protein (H6S33_011528) and ubiquitin-conjugating enzyme (H6S33_003916) were upregulated at 20–30 °C.

## 4. Discussion

### 4.1. Upregulation of Purine and Tyrosine Metabolism May Improve the Temperature Tolerance of Morchella Mycelia

As previously reported, the purine nucleotide and tyrosine metabolism pathways are crucial for the development of *Morchella* mycelia [23]. In this study, through a KEGG enrichment analysis, the DEGs associated with the 5 °C (vs. 20 °C) and 10 °C (vs. 20 °C) culture temperatures were significantly enriched in the purine metabolism pathway, while the DEGs associated with the 25 °C (vs. 20 °C) and 30 °C (vs. 20 °C) culture temperatures were significantly enriched in the tyrosine metabolism pathway (Figure 2B), which indicated that purine and tyrosine metabolisms have different responses to temperature. Purine nucleotides play important roles in determining genetic composition (DNA and RNA), energy metabolism (ATP and GTP), and signal transduction (cAMP) and provide the necessary coenzymes (NAD^+^, NADP^+^, and coenzymes A) to promote cell survival and proliferation [26,27]. The biosynthesis and catabolism of purine nucleotides are involved in the most basic cellular and biochemical processes and are essential for primary metabolism, secondary metabolism, and gene expression, which play important roles in the growth and development of fungi [28,29]. According to the GSEA, peptide metabolism, translation, and cytoplasmic translation were enriched at 5 °C, 10 °C, and 15 °C compared to 20 °C (Figure 3A–C), indicating that the stress caused by low temperatures on *Morchella* mycelia mainly affects transcription and translation processes. WGCNA also revealed that the ME blue module was enriched with many ribosomal protein- and purine catabolism-related genes, including inosine-5′-phosphate lactate dehydrogenase (IMPDH), xanthine dehydrogenase (XDH), and hypoxanthine guanine phosphoribosyl transferase (HGPRT) (Figure 5B). These genes encode rate-limiting enzymes that participate in regulating de novo synthesis, synthetic rescue, and degradation of purine nucleotides [30,31,32], which are upregulated with decreasing culture temperature (Figure 5B), further indicating that low temperatures induce the expression of genes related to purine nucleotide metabolism pathways to regulate the homeostasis of purine nucleotides, thereby improving stress resistance and maintaining mycelial development.

The tyrosine metabolism pathway is the starting point for the production of various naturally occurring compounds with diverse structures in plants. Previous metabolomic analysis revealed many metabolites involved in the tyrosine metabolism pathway among the primary metabolites; the tyrosine metabolism pathway was the most abundant metabolic pathway among the differentially accumulated metabolites of *Morchella* mycelia [33]. Similarly, the enrichment of the tyrosine pathway during mycelial development at high temperatures demonstrated that various active substances related to tyrosine metabolism may be produced to increase the heat resistance of mycelia and maintain their growth. Moreover, tyrosine metabolism participates in the biosynthesis of phenolic metabolites, which, in turn, affects the flavor of *Morchella* [26]. Flavor genes of *Morchella* that were negatively regulated in response to changes in temperature were also queried in the RNA sequence results (Figure 5B). Tyrosine metabolism is an important pathway for melanin biosynthesis in fungal cell walls and the cytoplasm, and genes encoding browning-related enzymes are involved in tyrosine metabolism [34,35]. Browning is considered a protective mechanism against cell integrity damage and can protect the body from ROS-induced DNA damage [36]. In the present study, the pigment deposition phenomenon observed in *Morchella* mycelia exposed to 25 °C and 30 °C (Figure S1A) may be related to the role of tyrosine metabolism in repairing cell damage caused by high-temperature stress.

### 4.2. Changes in Oxidative Stress and Energy Metabolism in Morchella Mycelia under High-Temperature Conditions

Notably, the WGCNA results demonstrated that the enrichment of the ME turquoise module was related to oxidative dehydrogenation and the catabolism of glucose under high culture temperatures (Figure 5A). With an increase in culture temperature, a large set of genes involved in oxidative dehydrogenation, such as alpha-ketoacid dehydrogenase kinase (H6S33_000063), sphingolipid fatty acid hydroxylase (H6S33_000109), mitochondrial dicarboxylate carrier protein (H6S33_000464), putative Fe-containing alcohol dehydrogenase (H6S33_000501), aldehyde dehydrogenase (H6S33_000904, H6S33_011849), oxidoreductase (H6S33_011977), and pyruvate decarboxylase (H6S33_012945), were upregulated in *Morchella* mycelia (Figure 5A), indicating that high-temperature stress leads to excessive accumulation of ROS in mycelial cells. As a cellular second messenger, abnormally accumulated ROS can oxidize macromolecules such as DNA, lipids, and proteins, thereby causing damage to organisms [14,37]. Similarly, the enrichment of the autophagy of mitochondria, regulation of cell morphogenesis, and piecemeal microautophagy of the nucleus at 25 °C (vs. 20 °C) also supported the disruption of the heat stress response in *Morchella* mycelia (Figure 3D), which was consistent with the positive regulation of mycelial pigment deposition by temperature (Figure S1A). Moreover, various studies have documented the regulatory activity of antioxidant enzymes, including APX, SOD, CAT, POD, and GSH, for the removal of reactive oxygen species and reducing the damage to cells in response to heat stress [38,39,40,41]. Consistent with the findings of previous reports, the results of the present study also suggested that the expression of the SOD and GSH genes in mycelia was upregulated to cope with ROS damage under high temperatures, with a similar variation trend observed for enzyme activity levels (Figure 6C and Figure 7). In addition, heat shock proteins serve as the main mitigators of cellular stress, and high temperatures significantly upregulated the expression levels of HSP12, HSP20, and HSP98/104 family proteins (Figure 6B). These results further indicated that high temperatures cause more severe oxidative damage to *Morchella* mycelia than low temperatures.

In addition to oxidative damage, high temperatures can disrupt the balance of central energy metabolism in cells [13,42,43]. Exposure of *Morchella* mycelia to high-temperature stress led to an increase in the expression of genes associated with energy metabolic activity, mostly related to monosaccharide metabolic, carbohydrate catabolic, and hexose metabolic processes (Figure 3E), indicating that fungi may compensate for the energy required for responding to high-temperature stress and the extra energy used to deliver stress responses by increasing energy metabolism [17]. *Morchella* species are saprophytic fungi, and they mainly derive carbon from carbohydrate catabolism, utilizing the carbon sources produced by degrading cellulose, hemicellulose, and starch. Carbohydrate transporters are mainly used for nutrient absorption or immune stress response [44]. When fungi are subjected to temperature stress, they can produce extracellular hydrolases to utilize different carbon and nitrogen sources to meet metabolic requirements [45]. The MFS monosaccharide transporter H6S33_000450 was identified among the module I genes, which is consistent with the findings of previous reports showing that major facilitator superfamily (MFS) proteins are involved mainly in energy metabolism [17]. Fungal cell mitochondria generate a large amount of energy in the form of ATP for proliferation and delayed death. Compared to the production of 36 ATPs per molecule of glucose through oxidative phosphorylation, the efficiency of glycolysis is lower, with only 2 ATPs generated. Notably, the increase in metabolism caused by high temperatures should maintain oxidative phosphorylation, allowing cells to generate enough energy to quickly resume growth [46]. However, in the present study, genes related to glycolysis, including glyceraldehyde 3-phosphate dehydrogenase (H6S33_011274) and pyruvate decarboxylase (H6S33_012945), were found to be positively regulated by temperature (Figure 5A). This may be because high temperatures cause damage to mitochondria and autophagy, leading to a decrease in energy production efficiency, which, in turn, leads to the generation of intermediate metabolites through increased glycolysis to help organisms adapt to ROS damage [42]. This also suggests that as the temperature increases, mycelial cells may no longer be able to recover normal growth. Previous reports have shown that fungi respond to high-temperature-induced ROS damage by shifting from mitochondrial respiration to glycolysis [47].

## 5. Conclusions

Integrating the findings of a mycelial phenotype analysis and transcriptome sequencing led to a deeper understanding of the temperature adaptation mechanisms in *Morchella* species and strains. The growth rate and colony morphology of mycelia from 13 *Morchella* strains at different cultivation temperatures indicated that 15–20 °C was the optimal temperature range for mycelial growth. A transcriptome analysis further revealed that the changes in the DEGs (vs. 20 °C), GO classifications, and metabolic processes associated with mycelia in response to low and high temperatures were not the same. Low temperatures did not cause irreversible damage to cells, with the response to this stress occurring mainly through ribosomal synthesis and purine metabolism. The effect of high-temperature stress on mycelia was significantly greater than that of low-temperature stress. Mycelial cells responded to high-temperature damage by enriching tyrosine metabolism, regulating energy metabolism pathways, and producing antioxidant enzymes. These research results contribute to a better understanding of the temperature tolerance mechanism of mycelia and provide novel insights for optimizing the cultivation and breeding of different *Morchella* species and strains.

## Figures and Tables

**Table 1:** Gene set enrichment analysis (GSEA).

Sample	Name	Size	Normalizing Enrichment Score	*p* Value	Leading Edge
5 °C vs. 20 °C	GO_0006518-peptide metabolic process	279	4.83	0	tags = 66%, list = 17%, signal = 75%
	GO_0043604-amide biosynthetic process	304	4.77	0	tags = 64%, list = 16%, signal = 73%
	GO_0044391-ribosomal subunit	140	4.72	0	tags = 75%, list = 13%, signal = 85%
10 °C vs. 20 °C	GO_0006412-translation	248	4.03	0	tags = 69%, list = 18%, signal = 81%
	GO_0005840-ribosome	162	3.96	0	tags = 79%, list = 18%, signal = 94%
	GO_0002181-cytoplasmic translation	163	3.94	0	tags = 74%, list = 18%, signal = 87%
15 °C vs. 20 °C	GO_0006412-translation	248	3.37	0	tags = 65%, list = 22%, signal = 80%
	GO_0002181-cytoplasmic translation	163	3.35	0	tags = 72%, list = 23%, signal = 91%
	GO_0005840- ribosome	162	3.25	0	tags = 68%, list = 22%, signal = 85%
25 °C vs. 20 °C	GO_0000422-autophagy of mitochondrion	18	1.95	0	tags = 67%, list = 24%, signal = 88%
	GO_0022604-regulation of cell morphogenesis	57	1.92	0	tags = 42%, list = 18%, signal = 51%
	GO_0034727-piecemeal microautophagy of the nucleus	26	1.86	0	tags = 62%, list = 28%, signal = 85%
30 °C vs. 20 °C	GO_0005996-monosaccharide metabolic process	44	2.11	0	tags = 57%, list = 18%, signal = 69%
	GO_0016052-carbohydrate catabolic process	67	2.08	0	tags = 49%, list = 18%, signal = 60%
	GO_0019318-hexose metabolic process	31	2.04	0	tags = 58%, list = 18%, signal = 71%

## Data Availability

Data are contained within the article.

## Supplementary Materials

The following supporting information can be downloaded at: https://www.mdpi.com/article/10.3390/jof10030178/s1, Figure S1: Morphological features of *Morchella* mycelia (A) and mycelial branches (B) in solid media at different culture temperatures.; Figure S2: Cluster analysis (A) and PCA (B) of intergroup differences at various culture temperatures; Figure S3: Numbers of differentially expressed genes (DEGs) in different treatment groups (vs. 5 °C or 20 °C). (A) Column chart of DEGs for each treatment group. (B) Volcano maps of upregulated and downregulated genes in different treatment groups. The red squares are upregulated genes, the blue squares are downregulated genes, and the gray areas are genes with not significant difference in expression; |log2 FC| ≥ 1, *p* < 0.05; Figure S4: Analysis of the ubiquitin-proteasome system gene expression in *Morchella* mycelia under different temperature treatments. Different lowercase letters indicate a significant difference between different temperatures (*p* < 0.5); Table S1: Sources of *Morchella* strains; Table S2: Mycelial growth rate of *Morchella* strains at different culture temperatures; Table S3: Sequencing statistics of the M37 strain transcriptome.
