# Supplementary material for: Comparative Transcriptome Profiles of the Response of Mycelia of the Genus Morchella to Temperature Stress: An Examination of Potential Resistance Mechanisms
Source: J Fungi (Basel). 2024 Feb 27;10(3):178. doi: 10.3390/jof10030178 (PMC10971721; doi:10.3390/jof10030178)
Supplement: Supplementary file 1 [file jof-10-00178-s001.zip › jof-2838568-supplementary.pdf]

Supporting Information

Table S1 Sources of *Morchella* strains

Strain name	Variety	Source	the ITS Sequencing Information
M6	M.*sextelata* M.Kuo	Hebei	TATTAAGAACACACAGACAGGGCTGCTATAGGGGCC<br>GGCAGGGCTAGTAGCTTTACGTTGTTGAACGTCCTGT<br>TTGGACCCGTTGGCAGCCCCCATCTAAACCCTCTGCG<br>TACCTGTCCCCCCTTGCTTCCCCCGGCACCTCGCTGG<br>GGGGAGGAACAACAACCAAAACTCTTTGTGAACAAA<br>CAGACGTCAGAATTACAAAAACAAAAAAAAGTTAA<br>AACTTTCAACAACGGATCTCTTGGTTCCCACATCGAT<br>GAAGAACGCAGCGAAATGCGATAAGTAATGTGAATT<br>GCAGAATTCAGTGAATCATCGAATCTTTGAACGCAC<br>ATTGCGCCCCCTGGTATTCCGGGGGGCATGCCTGTTC<br>GAGCGTCATAAAAACCTCCTCCCCCTTCGGGTTTGAT<br>TACTATCGTTGGGGGGTTTTGGCCTAATGGGATAGC<br>GATTGGCAATTAGTTTCCCAATGTCCTAAATAGACGT<br>AGACCCGCCTCCAGATGCGACAGCACCGAGGCCATC<br>AACCGTGGAGTTATGGGATATATAGGCTTGCAGTAA<br>AATGCTCACCTTTCTCCATACGCCGATGGCACACCGG<br>TCGCAGTTGCGGGCGTAAATTGGAGTCCTCTTCAGG<br>ACCCTCGTGGCCTAGCATCCACCATACATAATTTGAC<br>CTCGGATCAGGTAGGGATACCCGCTGAACTTAAGCA<br>TATCATAAACCGGGAAGAGAAAAAAAAA
M36	M.*sextelata* M.Kuo	Hubei	TTTCCTCCGCTTATTGATATGCTTAAGTTCAGCGGGT<br>ATCCCTACCTGATCCGAGGTCAAATTATGTATGGTGG<br>ATGTTAGTCCACGAGGGTCCTGAAGAGGACTCCAAT<br>TTACGCCCGCAACTGCGACCGGTGTGCCATCGGCGT<br>ATGGAGAAAGGTGAGCATTTTACTGCAAGCCTATAT<br>ATCCCATAACTCCACGGTTGATGGCCTCGGTGCTGTC<br>GCATCTGGAGGCGGGTCTACGTCTATTTAGGACATT<br>GGGAAACTAATTGCCAATCGCTATCCCATTAGGCCA<br>AAACCCCCCAACGATAGTAATCAAACCCGAAGGGGG<br>AGGAGGTTTTATGACGCTCGAACAGGCATGCCCCC<br>CGGAATACCAGGGGGCGCAATGTGCGTTCAAAGATT<br>CGATGATTCACTGAATTCTGCAATTCACATTACTTAT<br>CGCATTTCGCTGCGTTCTTCATCGATGTGGGAACCAA<br>GAGATCCGTTGTTGAAAGTTTTAACTTTTTTTTGTTTT<br>TGTAATTCTGACGTCTGTTTGTTCACAAAGAGTTTTG<br>GTTGTTGTTCCTCCCCCCAGCGAGGTGCCGGGGGAA<br>GCAAGGGGGGACAGGTACGCAGAGGGTTTAGATGG<br>GGGCTGCCAACGGGTCCAAACAGGACGTTCAACAAC<br>GTAAAGCTACTAGCCCTGCCGGCCCCTATAGCAGCC<br>CTTTTCTGTGTGGTTCTTGGTAATGATCCTTCCGCAG<br>GTTCACCTACGGAAG

M37	M.*sextelata* M.Kuo	Hubei	GTACCTGCGGAAGGATCATTACCAAGAACCACACAG<br>AAAAGGGCTGCTATAGGGGCCGGCAGGGCTAGTAGC<br>TTTACGTTGTTGAACGTCCTGTTTGGACCCGTTGGCA<br>GCCCCCATCTAAACCCTCTGCGTACCTGTCCCCCCTT<br>GCTTCCCCCGGCACCTCGCTGGGGGGAGGAACAACA<br>ACCAAAACTCTTTGTGAACAAACAGACGTCAGAATT<br>ACAAAAACAAAAAAAAGTTAAAACTTTCAACAACG<br>GATCTCTTGGTTCCCACATCGATGAAGAACGCAGCG<br>AAATGCGATAAGTAATGTGAATTGCAGAATTCAGTG<br>AATCATCGAATCTTTGAACGCACATTGCGCCCCCTGG<br>TATTCCGGGGGGCATGCCTGTTCGAGCGTCATAAAA<br>ACCTCCTCCCCCTTCGGGTTTGATTACTATCGTTGGG<br>GGGTTTTGGCCTAATGGGATAGCGATTGGCAATTAG<br>TTTCCCAATGTCCTAAATAGACGTAGACCCGCCTCCA<br>GATGCGACAGCACCGAGGCCATCAACCGTGGAGTTA<br>TGGGATATATAGGCTTGCAGTAAAATGCTCACCTTTC<br>TCCATACGCCGATGGCACACCGGTCGCAGTTGCGGG<br>CGTAAATTGGAGTCCTCTTCAGGACCCTCGTGGCCTA<br>GCATCCACCATACATAATTTGACCTCGGATCAGGTA<br>GGGATACCCGCTGAACTTAAGCATATCAATAAGGCG<br>GAGGAA
M38	M.*sextelata* M.Kuo	Hubei	CTTTCGTAAAGGGTACCTGCGGAAGGATCATTACCA<br>AGAACCACACAGAAAAGGGCTGCTATAGGGGCCGG<br>CAGGGCTAGTAGCTTTACGTTGTTGAACGTCCTGTTT<br>GGACCCGTTGGCAGCCCCCATCTAAACCCTCTGCGT<br>ACCTGTCCCCCCTTGCTTCCCCCGGCACCTCGCTGGG<br>GGGAGGAACAACAACCAAAACTCTTTGTGAACAAAC<br>AGACGTCAGAATTACAAAAACAAAAAAAAGTTAAA<br>ACTTTCAACAACGGATCTCTTGGTTCCCACATCGATG<br>AAGAACGCAGCGAAATGCGATAAGTAATGTGAATTG<br>CAGAATTCAGTGAATCATCGAATCTTTGAACGCACA<br>TTGCGCCCCCTGGTATTCCGGGGGGCATGCCTGTTCG<br>AGCGTCATAAAAACCTCCTCCCCCTTCGGGTTTGATT<br>ACTATCGTTGGGGGGTTTTGGCCTAATGGGATAGCG<br>ATTGGCAATTAGTTTCCCAATGTCCTAAATAGACGTA<br>GACCCGCCTCCAGATGCGACAGCACCGAGGCCATCA<br>ACCGTGGAGTTATGGGATATATAGGCTTGCAGTAAA<br>ATGCTCACCTTTCTCCATACGCCGATGGCACACCGGT<br>CGCAGTTGCGGGCGTAAATTGGAGTCCTCTTCAGGA<br>CCCTCGTGGCCTAGCATCCACCATACATAATTTGACC<br>TCGGATCAGGTAGGGATACCCGCTGAACTTAAGCAT<br>ATCAATAGGCCGGAGGAAA
M39	M.*sextelata* M.Kuo	Hubei	GTACCTGCGGAAGGATCATTACCAAGAACCACACAG<br>AAAAGGGCTGCTATAGGGGCCGGCAGGGCTAGTAGC<br>TTTACGTTGTTGAACGTCCTGTTTGGACCCGTTGGCA<br>GCCCCCATCTAAACCCTCTGCGTACCTGTCCCCCCTT<br>GCTTCCCCCGGCACCTCGCTGGGGGGAGGAACAACA<br>ACCAAAACTCTTTGTGAACAAACAGACGTCAGAATT<br>ACAAAAACAAAAAAAAGTTAAAACTTTCAACAACG<br>GATCTCTTGGTTCCCACATCGATGAAGAACGCAGCG<br>AAATGCGATAAGTAATGTGAATTGCAGAATTCAGTG<br>AATCATCGAATCTTTGAACGCACATTGCGCCCCCTGG<br>TATTCCGGGGGGCATGCCTGTTCGAGCGTCATAAAA<br>ACCTCCTCCCCCTTCGGGTTTGATTACTATCGTTGGG

			GGGTTTTGGCCTAATGGGATAGCGATTGGCAATTAG<br>TTTCCCAATGTCCTAAATAGACGTAGACCCGCCTCCA<br>GATGCGACAGCACCGAGGCCATCAACCGTGGAGTTA<br>TGGGATATATAGGCTTGCAGTAAAATGCTCACCTTTC<br>TCCATACGCCGATGGCACACCGGTCGCAGTTGCGGG<br>CGTAAATTGGAGTCCTCTTCAGGACCCTCGTGGCCTA<br>GCATCCACCATACATAATTTGACCTCGGATCAGGTA<br>GGGATACCCGCTGAACTTAAGCATATCAATAGCCGG<br>AGGAAC
M4	M.*sextelata*<br>M.Kuo	Shanxi	GAGGTTTCCGAAAACACCCTAATTTGGCTGCTATAC<br>GGTCCGGGTGGATTATACCGTGTTGTTTGTGCAGCCC<br>CCTGTTTTGACCGGTTGGGGCCCCCGCCTTTGCCCCC<br>CGGGCACCTGCCCCCCGTAGATCCCCCCGGCACCTCT<br>GTGGGGGGAGGAACAGATGAGTTGATCTTTGCAATC<br>AATTGACCTTTCAATTAAGGATCTCTTGGTTCCTGCA<br>TCTTTGAATAACGCATCGCATGGTTCCCACGAATGTG<br>AATAACAAAATTCAGTGAATTATCCAGTCTTTTTGGC<br>AATTGAGCCCCTTGGGCCTCTTCCTTCTATGATTGCT<br>CGAATGGGTTGCTGCTCTCGACGCTGGGTTGTGGTCC<br>GTTTACCCTTCCCCCCCTCTCGAGGCTGATACCCATC<br>TTTGAAGGGTTGTCCCGAGTTGGATAATACTATCGTC<br>TCGAGCTTTCGCACTGTGCTAGTTAGATTGATAGGCG<br>TTGTGCAGATGTATTAAAAAATGTTTATCACAAAGCT<br>GAGCAAATTTGAGGTAATGATGGCTCCCTAAAAATA<br>TTTTCATTTTATTTTTTGAGAAAAATTTTTACCGTTGT<br>GGGCGTGTATCGGGGCTACATCCATCCTCTCTATAAA<br>TTCTGGTTGTTTCGACGGGCCCCCATGCTGAGCCGGG<br>GGGGTGCTTTGCCCCTAGCCCCGCCGCGGTATCCCCA<br>GAAAACGGTGGAAAAGTAATTCGTGGGTACAATGAA<br>ATGCGATAAAAGGGATGCTTTATGGGGCGGCTGGTT<br>TGCATTCTTACCTAGTTGCAAATTCGGCTTGTTCATT<br>TGCCTCCAGTTAAACCTCGGCATTGTTTCTTGGTTTC<br>TCCGACCTTCTTTTGGGGTGGCGGACGACCTAGATCT<br>TTGGGAGCTAATAGACGTTATGACTTTAATAAGATA<br>GATTTTTTCCTTCTTCACTCCTGTGTGATGTCGTAGTC<br>ATATTGTAAACTGCTGTCGTGCACACATTCTTGCTCA<br>AGTATCACCCGTAGACACCTAACATACAACATACAT<br>GGTCCTCGACTGCTCGCATCAGGTCTGCCGTCGATTG<br>CAAAGCATCCAGTCGACTCATAACG
M15	M.*importun*<br>*a*<br>M.Kuo,<br>O'Donnell&<br>T.J.Volk	Sichuan	ACGGCATCCCTACCTGATCCGAGGTCAATTGTGTATG<br>GTGGATGCTAGGCCACGAGGGTCCTGAAAAAAGGGC<br>TCCAATTTACGCCCGCAACTGCGACCGGTGTGCCATC<br>GGCGTATGGAGAAAGGTGAGCATTTTACTGCAAGCC<br>TATATATCCCATAACTCCACGGTTGATGGCCTCGGTG<br>CTGTCGCATCTGGAGGCGGGTCTACGTCTATTTAGGA<br>CATTGGGAAACTAATTGCCAATCGCTATCCCATTAG<br>GCCAAAACCCCCCAACGATAGTAATCAAACCCGATG<br>GGGGAGGAGGTTTTTATGACGCTCGAACAGGCATGC<br>CCCCCGGAATACCAGGGGGCGCAATGTGCGTTCAAA<br>GATTCGATGATTCACTGAATTCTGCAATTCACATTAC<br>TTATCGCATTTCGCTGCGTTCTTCATCGATGTGGGAA<br>CCAAGAGATCCGTTGTTGAAAGTTTTAACTTTTTTGT

			TTTGTTATGATTCTGACGTCGGCTTGTTCACAAAGAG<br>TTTTGGTTGTTGTTCCTCCCCCCAGCGGGTAGCCGGG<br>GGAAGCAAGGCGGGACAGGTACGCAGAGGGTTTAG<br>ATGGGGGCGGCTCCGGGTCCGGCCAGGACGTTCAAC<br>AACGTAAAGCTACTAGCCCTGGTGGCCCCTCGGCTG<br>CCCTTTTCTGTGTGGTTCTTGGTAATGATCCTTCCGC<br>AGGTTCACCCTACGGAA
M24	M.*sextelata* M.Kuo	Qinghai	TAAATTTGGGTTTCCTAACCTTGATCCGAGGTCAAAT<br>TATGTATTGGTGGATGCTAGGCCACGAGGGTCCTGA<br>AGAGGACTCCAATTTACGCCCGCAACTGCGACCGGT<br>GTGCCATCGGCGTATGGAGAAAGGTGAGCATTTTAC<br>TGCAAGCCTATATATCCCATAACTCCACGGTTGATGG<br>CCTCGGTGCTGTCGCATCTGGAGGCGGGTCTACGTCT<br>ATTTAGGACATTGGGAAACTAATTGCCAATCGCTAT<br>CCCATTAGGCCAAAACCCCCCAACGATAGTAATCAA<br>ACCCATAAGGGGGAGGAGGTTTTATGACGCTCGAA<br>CAGGCATGCCCCCCGGAATTTTTTTTTTTTTTTTT
M41	M.*sextelata* M.Kuo	hybridization	*M.sextelata* hybridization
M42	M.*sextelata* M.Kuo	hybridization	*M.sextelata* hybridization
M47	M.*sextelata* M.Kuo	hybridization	*M.sextelata* hybridization
M32	M.*septimelata* M.Kuo	Sichuan	CTCCTTCCGTAGGGGAACCTGCGGAAGGATCATTAC<br>CAAGAACCACACAGAAAAGGGCAGCCGAGGGGCCA<br>ACCAGGGCTAGTAGCTTTACGTTGTTGAACGTCCTGG<br>AACGGACCCGGAGCCGCCCCCATCTAAACACTCTGC<br>GTACCCATCCCACCTTGCTTCCCCCGGCCATCCGCTG<br>GGGGGAGGAACAACAACCAAAACTCTTTGTGAAGA<br>AACAGACGTCAGAATCATAACCAAAAAAAAGTTAA<br>AACTTTCAACAACGGATCTCTTGGTTCCCACATCGAT<br>GAAGAACGCAGCGAAATGCGATAAGTAATGTGAATT<br>GCAGAATTCAGTGAATCATCGAATCTTTGAACGCAC<br>ATTGCGCCCTCTGGTATTCCGGGGGGCATGCCTGTTC<br>GAGCGTCATAAAAACCTCCTCCCCCTTCGGGTTTGAT<br>TACTATCGTTGGGGGGTATTGGCCTACTGGGAAAGC<br>GATTTGGCAATTGCCTTCCCACTGTCCTAAATACACT<br>TAGACCCGCCTCCAGATGCGACAGCACCGAGGCCAT<br>CAACCGTGGAGTTATGGAATACCGTTCTCCACACGC<br>CGATGGCAAACCGGTCGCAGTTGCGGGCGTAAATTG<br>GAGCCCTCTTCAGGACCCTCGTGGCCTAGCATCCACC<br>ATACATATTTTGACCTCGGATCAGGTAGGGATACCC<br>GCTGAACTTAAGCATATCAATAAGCGGGAGGAA

M33	M.*septimelata* M.Kuo	Sichuan	CTTCCCGTAGGGGGACCTGCGGAAGGATCATTACCA<br>AGAACCACACAGAAAAGGGCAGCCGAGGGGCCAAC<br>CAGGGCTAGTAGCTTTACGTTGTTGAACGTCCTGGA<br>ACGGACCCGGAGCCGCCCCCATCTAAACACTCTGCG<br>TACCCATCCCACCTTGCTTCCCCCGGCCATCCGCTGG<br>GGGGAGGAACAACAACCAAAACTCTTTGTGAAGAA<br>ACAGACGTCAGAATCATAACCAAAAAAAAGTTAAA<br>ACTTTCAACAACGGATCTCTTGGTTCCCACATCGATG<br>AAGAACGCAGCGAAATGCGATAAGTAATGTGAATTG<br>CAGAATTCAGTGAATCATCGAATCTTTGAACGCACA<br>TTGCGCCCTCTGGTATTCCGGGGGGCATGCCTGTTCG<br>AGCGTCATAAAAACCTCCTCCCCCTTCGGGTTTGATT<br>ACTATCGTTGGGGGGTATTGGCCTACTGGGAAAGCG<br>ATTTGGCAATTGCCTTCCCACTGTCCTAAATACACTT<br>AGACCCGCCTCCAGATGCGACAGCACCGAGGCCATC<br>AACCGTGGAGTTATGGAATACCGTTCTCCACACGCC<br>GATGGCAAACCGGTCGCAGTTGCGGGCGTAAATTGG<br>AGCCCTCTTCAGGACCCTCGTGGCCTAGCATCCACCA<br>TACATATTTTGACCTCGGATCAGGTAGGGATACCCG<br>CTGAACTTAAGCATATCAATAAGCGGAGGAA

Table S2 Mycelial growth rate of *Morchella* strains at different culture temperatures

	5℃ (mm/d)	10℃ (mm/d)	15℃ (mm/d)	20℃ (mm/d)	25℃ (mm/d)	30℃ (mm/d)
M6	2.55±0.02^{e}	7.03±0.00^{d}	9.90±0.02^{c}	13.99±0.00^{b}	15.75±0.04^{a}	9.67±0.3^{c}
M36	1.62±0.06^{f}	6.57±0.04^{e}	10.06±0.05^{c}	14.09±0.06^{b}	15.47±0.03^{a}	9.15±0.10^{d}
M37	2.74±0.04^{e}	6.88±0.02^{d}	9.44±0.61^{c}	14.37±0.04^{b}	15.57±0.07^{a}	9.53±0.38^{c}
M38	2.30±0.18^{f}	6.88±0.00^{e}	10.05±0.09^{c}	14.28±0.01^{b}	15.24±0.01^{a}	9.37±0.19^{d}
M39	3.33±0.07^{f}	8.25±0.00^{d}	11.59±0.04^{c}	14.43±0.02^{b}	17.11±0.07^{a}	8.51±0.05^{d}
M4	2.71±0.00^{e}	7.46±0.01^{e}	10.42±0.12^{b}	14.34±0.01^{a}	14.75±0.29^{a}	9.08±0.04^{c}
M15	1.11±0.00^{d}	7.25±3.16^{b}	8.70±0.08^{b}	11.54±0.31^{a}	11.81±0.16^{a}	5.48±0.04^{c}
M24	2.11±0.04^{f}	6.65±0.20^{e}	9.22±0.01^{c}	13.48±0.12^{b}	14.61±0.03^{a}	8.27±0.00^{d}
M41	2.30±0.01^{f}	6.75±0.06^{e}	10.68±0.03^{c}	15.69±0.10^{b}	16.54±0.02^{a}	9.77±0.13^{d}
M42	3.06±0.07^{f}	7.81±0.01^{e}	11.16±0.09^{c}	16.11±0.02^{b}	17.03±0.14^{a}	10.37±0.10^{d}
M47	2.56±0.16^{e}	7.50±0.13^{d}	10.56±0.34^{c}	15.71±0.07^{b}	16.88±0.21^{a}	9.89±0.51^{c}
M32	2.33±0.27^{d}	6.15±0.00^{c}	8.15±0.09^{b}	11.92±0.03^{a}	12.02±0.02^{a}	7.73±0.13^{b}
M33	2.48±0.00^{e}	6.00±0.00^{d}	8.40±0.00^{c}	11.90±0.02^{a}	11.85±0.02^{a}	9.03±0.08^{b}

The data are presented as the mean ± variance (n=10). Different lowercase letters indicate a significant difference between different temperatures for the same strain ($p < 0.5$).

Table S3 Sequencing statistics of the M37 strain transcriptome

Samples	Total reads	Total bases	Q20%	Q30%	GC%	Mapped Reads
M5-1	44,828,066	6,645,127,797	98.32	94.7	50.79	23,476,458
M5-2	48,116,918	7,102,736,430	98.27	94.57	50.76	25,040,044
M5-3	45,750,644	6,729,814,218	98.41	94.97	50.59	20,729,616
M10-1	45,314,956	6,707,400,340	98.53	95.3	50.67	22,154,481

M10-2	43,648,004	6,482,107,252	98.07	93.96	50.84	20,584,398
M10-3	47,740,460	7,110,811,813	98.14	94.15	50.26	22,905,872
M15-1	48,034,850	7,135,131,931	98.17	94.22	50.72	22,321,794
M15-2	45,477,298	6,756,366,324	97.07	91.27	50.65	20,555,738
M15-3	35,566,786	5,277,149,098	98.26	94.55	50.48	14,927,380
M20-1	43,179,844	6,409,317,025	98.09	94.05	49.91	21,529,470
M20-2	46,093,102	6,787,522,818	98.5	95.18	49.45	18,248,259
M20-3	48,813,138	7,211,468,456	98.41	94.95	49.75	20,106,131
M25-1	45,883,604	6,824,936,640	98.13	94.11	49.38	23,818,178
M25-2	47,069,594	7,004,368,605	98	93.69	49.67	33,080,510
M25-3	45,254,254	6,704,874,156	98.34	94.76	49.72	34,845,775
M30-1	46,633,778	6,915,881,579	98.3	94.64	49.92	29,062,170
M30-2	31,142,856	4,328,994,544	98.31	94.85	49.77	18,097,113
M30-3	46,526,824	6,880,456,766	98.46	95.09	49.74	24,640,605

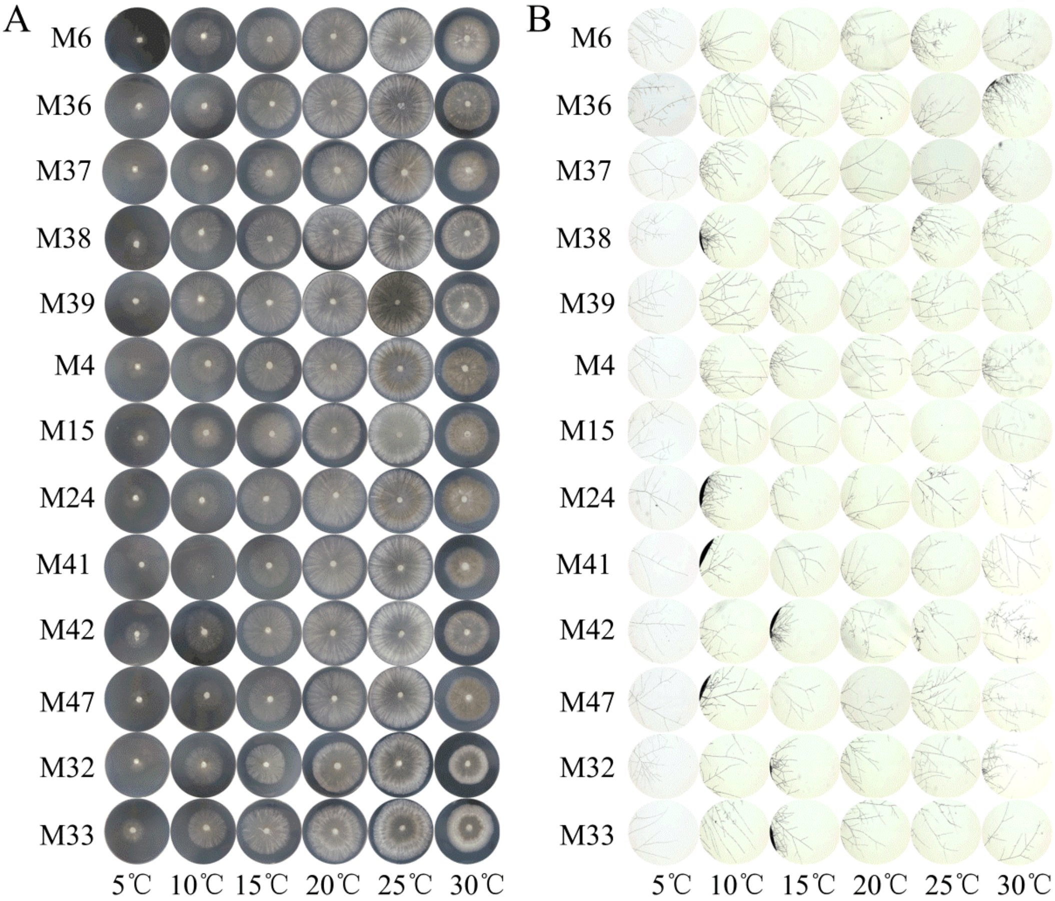

Fig. S1 Morphological features of *Morchella* mycelia (A) and mycelial branches (B) in solid media at different culture temperatures.

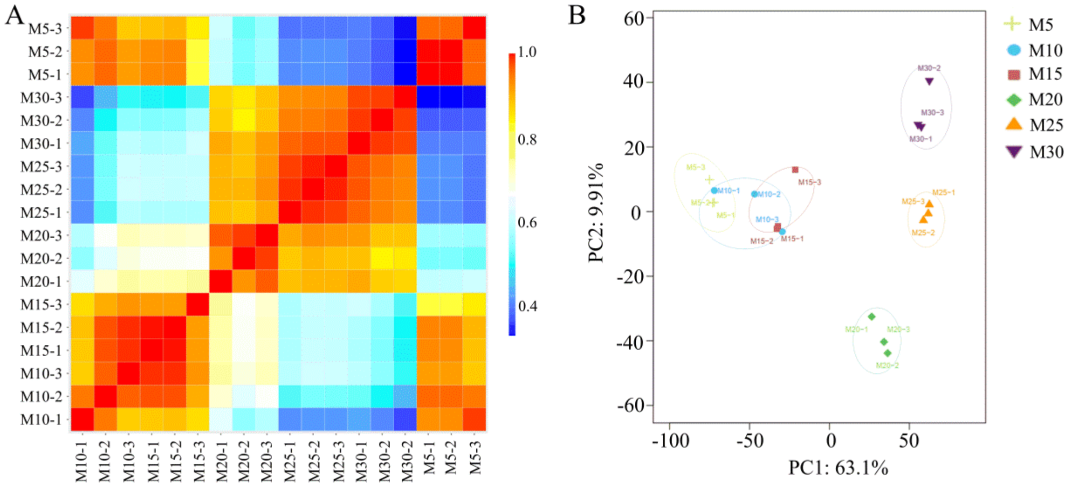

Fig. S2 Cluster analysis (A) and PCA (B) of intergroup differences at various culture temperatures.

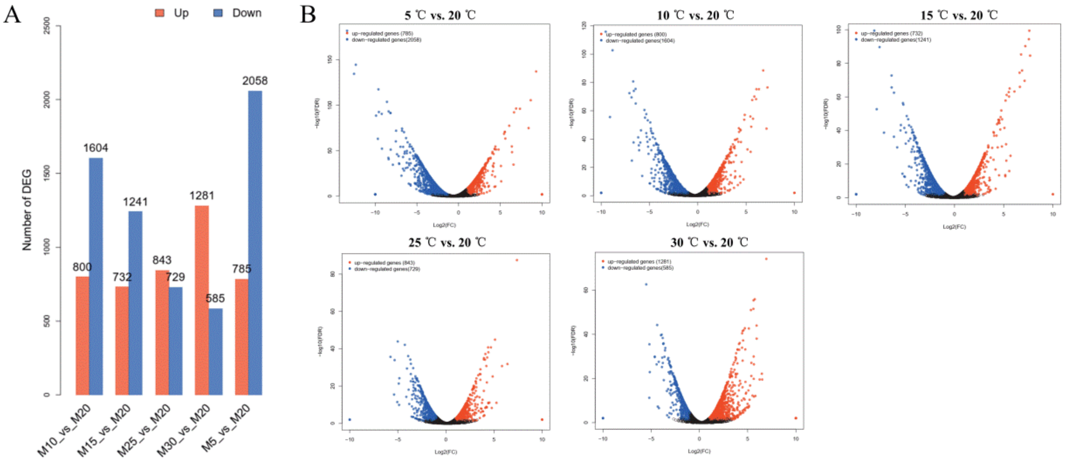

Fig. S3 Numbers of differentially expressed genes (DEGs) in different treatment groups (vs. 5 ℃ or 20 ℃). (A) Column chart of DEGs for each treatment group. (B) Volcano maps of upregulated and downregulated genes in different treatment groups. The red squares are upregulated genes, the blue squares are downregulated genes, and the gray areas are genes with not significant difference in expression; |log2 FC| ≥ 1, $p < 0.05$.

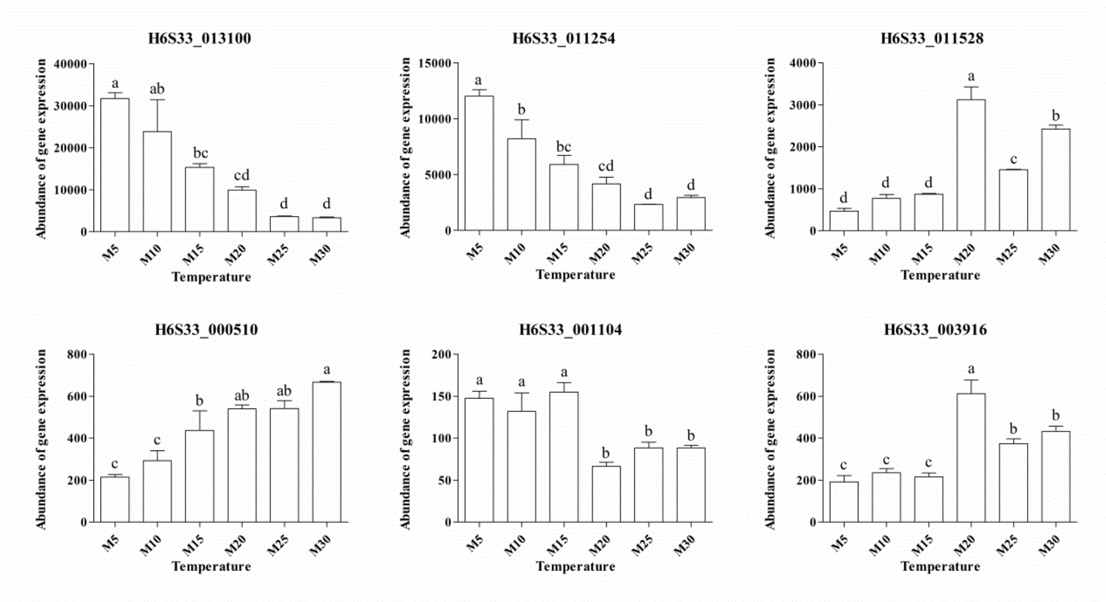

Fig. S4 Analysis of the ubiquitin-proteasome system gene expression in *Morchella* mycelia under different temperature treatments. Different lowercase letters indicate a significant difference between different temperatures ($p < 0.5$).
